# Intersectional analysis of social determinants of health and their association with mortality in patients with multimorbidity

**DOI:** 10.7189/jogh.14.04229

**Published:** 2024-10-18

**Authors:** Aida Moreno-Juste, Clara Laguna-Berna, Beatriz Poblador-Plou, Amaia Calderón-Larrañaga, Julián Librero, Cristina Lozano-Hernández, Alejandro Santos-Mejías, Marcos Castillo-Jimena, Antonio Gimeno-Miguel, Luis A Gimeno-Feliú

**Affiliations:** 1 EpiChron Research Group, Aragon Health Sciences Institute (IACS), IIS Aragón, Miguel Servet University Hospital, Zaragoza, Spain; 2 Illueca Primary Care Health Centre, Aragon Health Service (SALUD), Zaragoza, Spain; 3 Network for Research on Chronicity, Primary Care, and Health Promotion (RICAPPS), Institute of Health Carlos III (ISCIII), Madrid, Spain; 4 Ageing Research Centre, Department of Neurobiology, Care Sciences and Society, Karolinska Institute and Stockholm University, Stockholm, Sweden; 5 Stockholm Gerontology Research Centre, Stockholm, Sweden; 6 Unidad de Metodología, Navarrabiomed-HUN-UPNA, Pamplona, Spain; 7 Research Unit, Primary Health Care Management Madrid, Madrid, Spain; 8 Biosanitary Research and Innovation Foundation of Primary Care (FIIBAP), Madrid, Spain; 9 Faculty of Health, Universidad Camilo José Cela, Madrid, Spain; 10 Department of Pharmacology and Paediatrics, School of Medicine, University of Malaga (Universidad de Málaga), Málaga, Spain; 11 Group C-08 Biomedical Research Institute of Málaga -IBIMA-, Málaga, Spain; 12 Primary Care Health Centre Campillos, Northern Málaga Integrated Healthcare Area, Andalusian Health Service, Campillos, Málaga, Spain; 13 University of Zaragoza, Zaragoza, Spain; 14 San Pablo Primary Care Health Centre, Aragon Health Service (SALUD), Zaragoza, Spain; * Joint senior authorship.; **Background** We aimed to analyse the association between social determinants of health (SDH) and mortality in patients with multimorbidity from an intersectional point of view.; **Methods** We conducted a retrospective observational study in the EpiChron cohort (Aragon, Spain), including all patients with two or more chronic conditions in 2015, who were followed up until 2020, to analyse all-cause mortality. Logistic regressions models were performed to analyse the likelihood of mortality across 24 intersectional strata defined by gender, migration status/length of stay, residence area and socioeconomic class. The area under the receiver operator characteristics curve was estimated to evaluate the discriminatory accuracy of mortality.; **Results** Nearly one in 10 people with multimorbidity died during the study period. The likelihood of mortality was higher in men, in people with lower annual gross income, and in those living in rural areas. The intersectional analysis showed that low-income migrant men with more than 15 years in Spain and living in rural settings had a 4.2 times higher risk of death than that observed in middle-high income, non-migrant, urban women (reference group). Women had a lower risk of mortality than men regardless of annual gross income, migration status and residence area. Migrants’ mortality risk varied depending on socioeconomic situation. All models had a large discriminatory accuracy, which increased across the intersectional analysis.; **Conclusions** There is a clear association between SDH and mortality in patients with multimorbidity. The intersectional approach used in this study revealed some interactions among these determinants, illustrating the social disadvantage that underlies the need to implement policies to promote equitable health promotion.

The progressive increase in the number of patients with multimorbidity (i.e. with two or more chronic diseases) represents a major global challenge for health systems and daily clinical practice, as well as for epidemiological research [1,2]. Recent evidence has highlighted the limitations of current health care systems for addressing the complex needs of patients with multimorbidity due to inadequate or absent attention to coexisting chronic conditions and failure to perform a comprehensive approach to their care [1,3].

Multimorbidity is considered an independent risk factor for mortality [2,4,5], especially in elderly people [6]. However, the predictors of mortality within the population with multimorbidity are not well described in the literature [2]. This higher risk of death has been related to an increase in the number of diseases, interactions between illnesses and drugs [7], fragmented care, higher rates of disability and frailty, and other factors like social support [4,8] and loneliness [4]. The relationships among these mechanisms are complex and are often moderated by sociodemographic characteristics [6].

The social determinants of health (SDH), defined as the social circumstances in which each person is born, grows, lives, works and gets older [9], influence the distribution of health inequalities and chronicity [1,3,10]. Many studies have analysed the relationship between multimorbidity and the SDH, finding that multimorbidity appears 10–15 years earlier in the most disadvantaged social classes [3,11,12]. However, results are sometimes inconsistent due to the fact that authors often fail to untangle the inter-relationship among these social factors [13] by focusing on one single social stratification dimension (i.e. gender, social class, age, race or migrant background) instead of using an intersectional approach [11,14,15].

The intersectionality theory focuses on the idea that social factors are interconnected rather than separate, creating overlapping and interacting systems of discrimination or disadvantage that accompany people in every social interaction [16–18]. This is why all these axes should be incorporated simultaneously into social analyses [11,15–19]. This theory represents a new way of understanding the complex nature of health inequities [11,16,20] by shifting the study of the SDH from an additive to a multiplicative interaction framework that must be assessed through a holistic biopsychosocial approach [11,18].

Although the role of the SDH in mortality has already been extensively investigated, less is known about its effect on mortality in patients with multiple long-term conditions who, due to their health status, could, therefore, be even more susceptible to this kind of inequality. The aim of this study was to analyse the association between the SDH (i.e. gender, migration status/length of stay, residence area and socioeconomic class) and mortality in patients with multimorbidity from an intersectional point of view.

## METHODS

### Study design and population

We conducted a retrospective observational study in the EpiChron cohort, which was created in 2011 to study the epidemiology of chronic diseases and multimorbidity in the Spanish region of Aragon. Aragon is an autonomous community located in northeast Spain with a reference population of 1.3 million inhabitants. It is characterised by a high geographical dispersion with low population density in rural areas and a few urban towns that concentrate the majority of the population. Aragon has a population slightly older than the national average, and migrant people represent around 13% of the total population.

The EpiChron links, at the patient level and in a pseudonymised way, the socio-demographic and clinical information for all public health care system users in the region. This open cohort, which is regularly updated, included information on 1 253 292 individuals of all ages at baseline (mean age is 44.2 years, 50.5% women, 11.9% migrants, 37.5% with multimorbidity, the mean burden of 1.7 chronic diseases and 4.3 drugs). The EpiChron integrates information from patients’ electronic health records (EHRs) from primary and hospital health care, pharmacy billing records, and users’ database, which includes socio-demographic data and information on the date (but not cause) of death. More information on the cohort profile regarding baseline information, data sources used, and details on data curation and linkage procedures has been published elsewhere [21]. The Clinical Research Ethics Committee of Aragon (CEICA) favourably evaluated the EpiChron Cohort Study (protocol number PI17/0024).

In this study, we included all patients from the EpiChron cohort who had multimorbidity (i.e. two or more diagnoses of chronic diseases) between 1 January and 31 December 2015 (enrolment period). To ensure completeness and accuracy of clinical information, we excluded individuals who had not been enrolled as users of the health care system for at least one year before 1 January 2015 and members with mutual insurance (1.06% of patients in Aragon) because information on their socioeconomic class-income was not available. Included patients were followed up from 1 January 2016 to 31 December 2020, their death or drop out from the users´ database, whichever occurred first.

### Study variables and data sources

In order to study the SDH, we extracted for each individual information on: gender, age (categorised as ≤44, 45–69, and ≥70 years), migrant status (migrant vs. native), length of residence in Aragon (short-term vs. long-term migrants with ≤15 or >15 years in the region, respectively) [11], residence area (urban, i.e. people living in municipalities that concentrate at least 80% of the population of the area, and rural, i.e. the rest [22]), and socioeconomic class. The socioeconomic class was measured using a proxy of annual gross income based on the prescription co-payment rate (low income<EUR 18 000, medium income EUR 18 000–100 000, and high income>EUR 100 000 [23,24]). Data on all-cause mortality was integrated for the follow-up period (1 January 2016 to 31 December 2020). Information on chronic conditions was extracted from patient’s EHRs. A chronic condition was defined as one that had been present for at least 12 months and meets one or both of the following criteria: 1) requires ongoing interventions using medical products, services, and/or special equipment, 2) entails limitations on self-care, independent living, and/or social interactions [25]. Diagnoses were originally coded using the first edition of the International Classification of Primary Care and subsequently mapped to the codes of the International Classification of Diseases, ninth edition, Clinical Modification [26]. The Clinical Classifications software, version ICD-9-CM (Agency for Healthcare Research and Quality, Rockville, Maryland, USA), was used to group all diagnoses into 226 clinically exclusive relevant categories [27] and the Chronic Condition Indicator software (Agency for Healthcare Research and Quality, Rockville, Maryland, USA) was used to classify them into chronic or non-chronic [28]. The list of 153 conditions considered chronic by this software was slightly adapted to facilitate the interpretation of results and 129 chronic conditions were finally identified and analysed in the study [25] (Table S1 in the **Online Supplementary Document**).

### Statistical analysis

The socio-demographic variables and the number of chronic diseases of the studied multimorbid population were described at baseline in 2015 using frequencies and percentages for categorical variables and means and standard deviations for continuous variables. Given that 71.4% of the population in the EpiChron cohort had a low annual gross income in 2019 [11], we jointly analysed the medium and high-income categories. Student’s *t* test or χ^2^ test were used, as appropriate, to assess the differences between men and women.

To develop the intersectional analysis, we created 24 intersectional strata by combining all possible categories of each variable: two for gender × three for migrant status/length of stay (native, short-term migrant, long-term migrant) × two for residence area × two for socioeconomic class (low or medium-high income). Then, an additive model was calculated through four consecutive logistic regression models [15,16,18] to estimate the likelihood of mortality against the different SDHs. The first regression model (Model 1) included gender, and the following models successively added annual gross income (Model 2), migrant status/length of stay (Model 3), and residence area (Model 4). Average marginal effects were used to estimate the average effect of the independent variables on the probability of occurrence of the contrast category of the dependent variable [29].

To test for multiplicative interactions, we built a logistic regression model that included all variables in Model 4 and all two-by-two and three-by-three interactions among them. Finally, to provide a visual representation of the intersectional dynamics among the studied SDHs, we developed a model including the 24 intersectional strata.

For each model, we quantified the area under the receiver operator characteristics (ROC) curve (AUC) and Nagelkerke’s pseudo R-squared to evaluate the discriminatory accuracy for mortality [18]. The AUC takes values between 0.5–1, where one indicates perfect discrimination and 0.5 means that the studied variables are not associated with mortality at all [15]. The discriminatory accuracy of the model was categorised according to AUC-ROC values as absent or very small (0.5–0.6), small (0.6–0.7), large (0.7–0.8), or very large (>0.8) [15].

All the analyses were conducted in STATA, version 12.0 (StataCorp LLC, College Station, Texas, USA). Statistical significance was set at *P*-value <0.05. Graphs were created using ‘ggplot2’ package [30] in R, version 4.3.1 (R Core Team, Vienna, Austria) [31].

## RESULTS

The clinical and demographical characteristics of the 652 201 patients with multimorbidity included in the study are shown in **Table 1**. Most of the patients were native (89.8%), older than 45 years (77.9%), and had a low annual gross income (69.1%). They had four diseases on average, and remarkably, almost one in 10 patients presented eight or more chronic conditions. In terms of gender differences, a greater proportion of women had a low income, who also presented with a slightly higher mean number of chronic diseases. In addition, there were more migrant women than men in absolute terms, especially from Eastern Europe and Latin America.

**Table 1:** Characteristics of the study population at baseline in 2015*

Characteristics	Men	Women	Total
Total	283 166 (43.4)	369 035 (56.6)	652 201(100.0)
Age in years, x̄ (SD)	55.39 (20.7)	56.39 (21)	55.95 (20.9)
Age group in years			
*0–44*	77 597 (27.4)	105 945 (28.7)	183 542 (28.1)
*45–69*	128 002 (45.2)	151 726 (41.1)	279 728 (42.9)
*≥70*	77 567 (27.4)	111 364 (30.2)	188 931 (29)
Migrant status			
*Non-migrant*	257 773 (91.0)	328 156 (88.9)	585 929 (89.8)
*Migrant*	25 393 (9.0)	40 879 (11.1)	66 272 (10.2)
Length of stay in Aragon in years			
*≤15*	22 072 (86.9)	36 885 (90.2)	58 957 (88.9)
*>15*	3321 (13.1)	3994 (9.8)	7315 (11.1)
Country of birth			
*Spain*	257 773 (91.0)	328 156 (88.9)	585 929 (89.8)
*Eastern Europe*	7387 (2.6)	12 491 (3.4)	19 878 (3.0)
*Asia*	1066 (0.4)	1189 (0.3)	2255 (0.4)
*North Africa*	3955 (1.4)	4390 (1.2)	8345 (1.3)
*Sub-Saharan Africa*	2641 (0.9)	1951 (0.5)	4592 (0.7)
*Latin America*	7956 (2.8)	17 949 (4.9)	25 905 (4.0)
*EU and North America*	2388 (0.9)	2909 (0.8)	5297 (0.8)
Residence area			
*Urban*	166 006 (58.6)	228 736 (62.0)	394 742 (60.5)
*Rural*	117 158 (41.4)	140 298 (38.0)	257 456 (39.5)
Annual gross income			
*Low*	174 751 (61.7)	275 777 (74.7)	450 528 (69.1)
*Medium*	106 718 (37.7)	92 021 (24.9)	198 739 (30.5)
*High*	1697 (0.6)	1237 (0.3)	2934 (0.4)
Number of chronic diseases, x̄ (SD)	3.92 (2.1)	4.36 (2.4)	4.17 (2.3)
Number of chronic diseases			
*2*	90 652 (32.0)	95 887 (26.0)	186 539 (28.6)
*3*	62 240 (22.0)	74 084 (20.1)	136 324 (20.9)
*4*	44 416 (15.7)	57 383 (15.5)	101 799 (15.6)
*5*	30 972 (10.9)	44 559 (12.1)	75 531 (11.6)
*6*	21 011 (7.4)	33 243 (9.0)	54 254 (8.3)
*7*	13 740 (4.9)	23 655 (6.4)	37 395 (5.7)
*≥8*	20 135 (7.1)	40 224 (10.9)	60 359 (9.3)
Death†	31 213 (11.0)	31 919 (8.7)	63 132 (9.7)
Loss to follow-up†	5259 (1.9)	8426 (2.3)	13 685 (2.1)

EU – European Union, SD – standard deviation, x̄ – mean

*Presented as n (%) unless specified otherwise.

†The numbers of deaths and losses to follow-up were calculated between 1 January 2016 and 31 December 2020

We observed that 9.7% of patients with multimorbidity died during the follow-up period. The two most prevalent diagnoses in dead patients were hypertension and disorders of lipid metabolism, followed by diabetes mellitus and other metabolic disorders in men and by genitourinary symptoms and osteoarthritis in women (Table S2 in the **Online Supplementary Document**).

Models 1–4 showed that being male, belonging to a low annual gross income group and living in rural areas were all factors associated with a higher likelihood of overall mortality (**Table 2**). On the contrary, being a short-term migrant was associated with a lower likelihood of mortality.

**Table 2:** Logistic regression models against five-year mortality*

	Model 1		Model 2		Model 3		Model 4	
**Characteristics**	**OR (95% CI)**	**AME (95% CI)**	**OR (95% CI)**	**AME (95% CI)**	**OR (95% CI)**	**AME (95% CI)**	**OR (95% CI)**	**AME (95% CI)**
Gender								
*Men*	1.84 (1.81, 1.88)	0.039 (0.038, 0.041)	1.93 (1.89, 1.97)	0.043 (0.042, 0.044)	1.93 (1.89, 1.97)	0.043 (0.042, 0.044)	1.92 (1.88, 1.96)	0.042 (0.041, 0.044)
*Women*	ref.	ref.	ref.	ref.	ref.	ref.	ref.	ref.
Annual gross income								
*Low*	NA	NA	1.53 (1.49, 1.57)	0.026 (0.025, 0.028)	1.54 (1.50, 1.58)	0.026 (0.025, 0.028)	1.51 (1.47, 1.55)	0.025 (0.024, 0.027)
*Middle-high*	NA	NA	ref.	ref.	ref.	ref.	ref.	ref.
Migrant status-residence length								
*Non-migrant*	NA	NA	NA	NA	ref.	ref.	ref.	ref.
*Migrant ≤15 y*	NA	NA	NA	NA	0.76 (0.70, 0.83)	–0.016 (–0.021, –0.011)	0.77 (0.71, 0.84)	–0.016 (–0.021, –0.011)
*Migrant >15 y*	NA	NA	NA	NA	0.89 (0.80, 1.00)	–0.007 (–0.014, 0.001)	0.91 (0.81, 1.02)	–0.006 (–0.013, 0.001)
Residence area								
*Urban*	NA	NA	NA	NA	NA	NA	ref.	ref.
*Rural*	NA	NA	NA	NA	NA	NA	1.09 (1.07, 1.11)	0.006 (0.004, 0.007)
AUC	0.8914		0.8926		0.8928		0.8928	
Nagelkerke R^2^	0.406		0.409		0.409		0.409	

AME – average marginal effects, AUC – area under the receiver operating characteristics curve, CI – confidence interval, NA – not applicable, OR – odds ratio, ref – reference

*ORs were obtained from four consecutive age-adjusted logistic regressions modelling the risk of mortality as a function of one to four independent variables.

We found significant two-by-two interactions between gender and annual gross income, gender and residence area, and migrant status-residence length and income (**Table 3**). Men had a higher risk of mortality than women, and this association was higher in men with low income (odds ratio (OR) = 2.85; 95% confidence interval (CI) = 2.73, 2.97) and in those living in rural areas (OR = 2.09; 95% CI = 2.04, 2.15). Long-term migrants (OR = 1.37; 95% CI = 1.34, 1.41) and non-migrants with low income (OR = 1.33; 95% CI = 1.17, 1.51) had a higher likelihood of mortality.

**Table 3:** Statistically significant interactions on mortality*

Two-by- two interactions	OR (95% CI)	*P*-value
Gender/annual gross income		
*Women/middle-high income*	ref.	
*Women/low income*	1.45 (1.39, 1.51)	<0.001
*Men/middle-high income*	1.80 (1.72, 1.89)	<0.001
*Men/low income*	2.85 (2.73, 2.97)	<0.001
*AUC*	0.8926	
Gender/residence area		
*Women/Urban*	ref.	
*Women/Rural*	1.20 (1.17, 1.23)	<0.001
*Men/Urban*	1.91 (1.86, 1.96)	<0.001
*Men/Rural*	2.09 (2.04, 2.15)	<0.001
*AUC*	0.8916	
Migrant status-residence length/income		
*Non-migrant/middle-high income*	ref.	
*Migrant >15 y/middle-high income*	0.66 (0.49, 0.89)	<0.001
*Migrant ≤15 y/low income*	0.96 (0.88, 1.06)	0.440
*Migrant ≤15 y/middle-high income*	1.05 (0.82, 1.34)	0.700
*Migrant >15 y/low income*	1.33 (1.17, 1.51)	<0.001
*Non-migrant/low income*	1.37 (1.34, 1.41)	<0.001
*AUC*	0.8880	

AUC – area under the receiver operating characteristics curve, CI – confidence interval, OR – odds ratio, ref – reference

*All models were adjusted for age in 2015.

**Figure 1:**
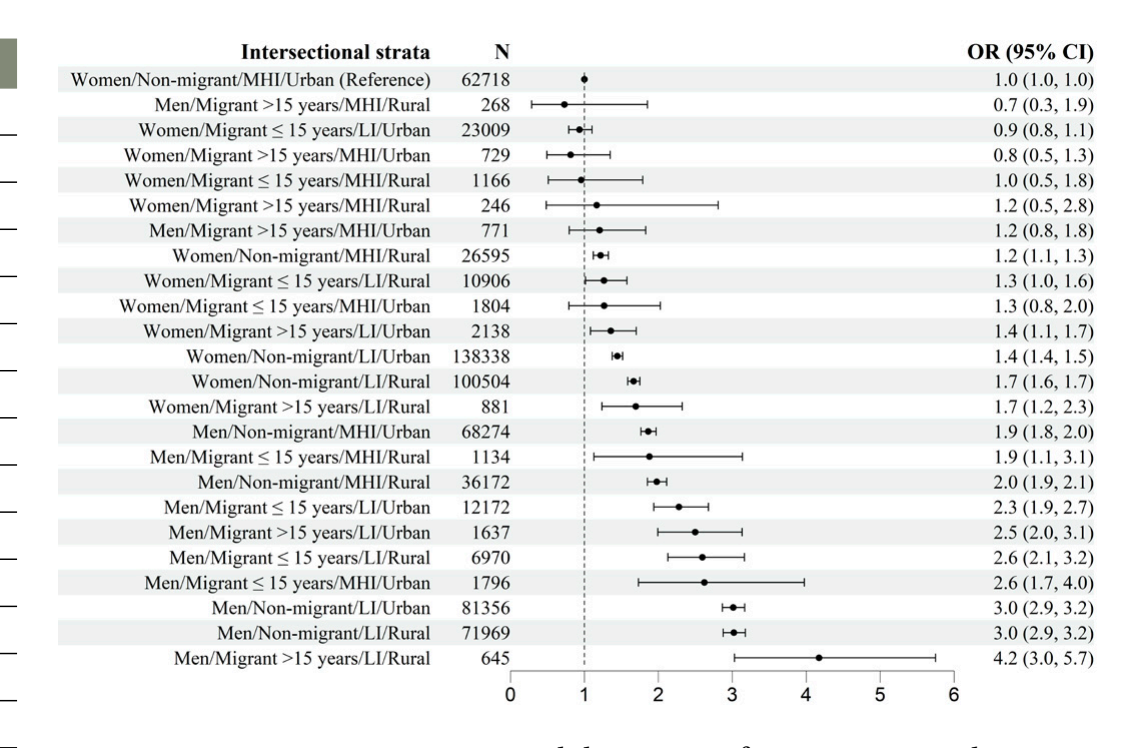
Logistic regression models against five-year mortality as a function of intersectional strata derived from all the social determinants of health analysed. Odds ratios with 95% confidence intervals were obtained from one single age-adjusted logistic regression model (Model 5), with gender, migrant status/length of stay, annual gross income, residence area, and their interactions in the form of intersectional strata as independent variables. The model area under the receiver operating characteristics curve (AUC) = 0.8929. CI – confidence interval, LI – low income, MHI – middle-high income, OR – odds ratio.

The intersectional gradient in the relationship between gender, migrant status/length of stay, annual gross income, and residence area is shown in **Figure 1**. The stratum with the highest likelihood of mortality was the group of rural long-term migrant men with low income, in whom the odds of mortality were 4.2 times higher compared with middle-high income, non-migrant, urban women (reference group). The intersectional analysis revealed a lower risk of mortality in women compared with men, regardless of annual gross income, migration status and residence area. More specifically, men with low income had at least twice the odds of mortality compared with the reference group. All models had a large discriminatory accuracy, which increased across the successive models developed.

## DISCUSSION

There is a clear association between the SDH and the likelihood of mortality in patients with multimorbidity. Being male, non-migrant, having low income, and living in a rural area are all factors associated with an increased mortality risk; however, the intersectional approach revealed potential synergies among exposures to the different SDH. The group of rural long-term migrant men with low annual gross income had the highest risk of mortality. In contrast, urban non-migrant women with middle- and high-income statuses represented the most advantaged group. Men had a higher risk of mortality than women regardless of annual gross income, migration status and residence area.

Patients with multimorbidity tend to have an increased risk of mortality compared with people with no chronic illnesses [4,5]. Even though multimorbidity prevalence is usually greater in women [11], we observed a more pronounced risk of mortality in men, as previously described [5,32].

This discrepancy has been attributed to various factors such as tobacco use, cardiovascular disease and social factors [5,32]. However, Halonen et al. observed that multimorbidity increased the risk of mortality in women but not in men and that cardiovascular diseases like diabetes and heart disease, and dementia were associated with higher mortality in both genders [2]. This phenomenon, known as the male-female health-mortality paradox, results from the fact that females live longer than males but spend a higher proportion of their total life expectancy in poorer health states [33].

Being male was consistently associated with an increased risk of mortality across the different analyses developed. The univariate analysis showed similar results to those observed in the two-by-two statistically significant interactions among SDH comparing gender, annual gross income and residence area. In contrast, the interaction between migrant status and income showed differences with the univariate analysis, in which short-term migrants had less mortality risk than non-migrants. These differences highlighted the need to study the interactions among SDH with an intersectional approach.

In our intersectional analysis, men continued to have a higher risk of mortality regardless of other SDH, but some differences were observed between the different strata resulting from the combinations of SDH. As happened in the univariate analysis, patients living in rural areas and with low socioeconomic status were at higher risk compared with patients living in urban areas with the same other determinants, except urban middle-high income short-term migrant men and women.

In general, low annual gross income was associated with a higher likelihood of mortality independently of the other SDH, with the exception of short-term migrants. Socioeconomic status has been associated with increased mortality [34,35], and it is considered a significant and independent predictor of mortality. However, a study in the UK Biobank cohort did not observe a significant statistical interaction between socioeconomic groups and the number of chronic diseases on mortality risk [36]. Along the same lines, Dugravot et al. found that socioeconomic status affected the risk of multimorbidity but did not increase mortality risk [8]. All in all, social inequalities are widely related to negative social and biological consequences that increase the risk of multimorbidity and mortality [11,16,37], and they are considered to be a risk factor for earlier death, independently of other risk factors such as diabetes, obesity, hypertension, smoking, alcohol consumption, or lack of physical activity [34,36,38]. In addition, the negative consequences of the social inequalities could be higher in migrants who are at risk of worse access to health care services due to work priorities, language barriers, low health literacy, or other accessibility problems [39,40].

The geographical context is also related to differences on individuals’ health status [37]. People living in rural areas or areas with less regional development are at risk of poor health outcomes [35,37], possibly because of poorer access to health care services [35]. However, further research is needed because of the inconsistency of the effect of rurality on multimorbidity and mortality rates [11,35,37,41].

Being a migrant has been described as a protective factor for the development of multimorbidity, although the length of stay of migrants has also to be considered. As the ‘healthy migration effect’ theory explains, migrants usually have a good level of health on arrival to the host countries, even higher than that of the host population. This phenomenon has also been described as the risk of mortality [40,42]. However, this effect diminishes with the length of stay, probably due to the adverse living conditions [11,39,40,42–44]. Still, in the two-by-two interaction analyses, we saw that long-term migrants with middle-high income had a lower risk of mortality than natives with the same income level. Indeed, mortality among migrants is influenced by other variables like gender, migrants’ region of origin, age at the time of migration [42], and other socioeconomic variables [40,42]. Moreover, the ‘salmon bias’ theory posits that there is a higher probability that migrants return to their country of origin when they grow old, retire, or become seriously ill, which would cause an underreporting of migrant mortality [39,42]. This might partially explain the differences found in the risk of mortality depending on migrants’ socioeconomic situation. The higher mortality risk seen among urban short-term migrants with middle-high income deserves further investigation.

The intersectional approach represents a valuable way of understanding the complex nature of health inequalities, and can help us to better map such inequalities and thus better illustrate patterns of oppression and social disadvantage [17]. It is, in essence, a way to understand and investigate people’s health in a holistic biopsychosocial manner [45]. This approach also helps us shift attention from individual risk factors to social power dynamics, reinforcing the importance of structural interventions that address social causes [3,9,20]. It can also help us to promote proportional universalism approaches that avoid ‘victim-blaming’, which is something we tend to do with individual approaches [16].

Further research is needed to explore the mechanisms underlying the interactions between the SDH and to develop local and national policies that take these factors into account [37]. More specifically, the implementation of measures to improve the socioeconomic conditions of the most unfavoured population groups, especially for multimorbid patients with low socioeconomic status and living in rural areas, is urgently warranted. The objective of these policies, which consider the intersectional influence of the SDH, should be to bring forward equitable health promotion.

### Strengths and limitations

The main strength of this study is its large-scale population-based nature, including virtually all patients with multimorbidity in the studied region. Moreover, we exhaustively analysed a total of 153 chronic diagnoses that were extracted from patients’ EHRs of both primary and hospital care for the definition of multimorbidity. Data from the EpiChron cohort undergo continuous quality controls, ensuring high levels of accuracy and reliability of the data for research purposes [21]. The main limitation of this study is inherent to its cross-sectional observational nature, which limits the establishment of causal relationships between exposures and the outcome analysed [11,15]. Another significant limitation is that the number of chronic conditions and all the independent variables were assessed only once at baseline, which prevents studying the changes produced in the study population during the follow-up. In addition, the cause of death was not available, which would have further enriched our intersectional insights. We also lacked information on some other psychosocial factors that may play an important role, like social support [4,37,46], loneliness [4], manual or non-manual work [37,46] or level of education [40,46]. We were not able to study outcomes related to frailty and disability, which are associated with increased risk of mortality [2,8], although some authors showed that this relation was not modified by socioeconomic status [8]. Stratification of the analyses by country of birth could be of interest to examine potential differences between countries [42], although it was outside the scope of the present study. Finally, some researchers have questioned the use of intersectional theory in a quantitative way and argue that this theory has been oriented towards qualitative research [11]; nonetheless, other authors have highlighted the importance of applying intersectional approaches in quantitative population health research.

## CONCLUSIONS

The SDH are clearly associated with mortality in patients with multimorbidity. Being male, non-migrant, having low income, and living in a rural area are factors associated with a higher likelihood of mortality. The intersectional approach helps us to reveal important interactions between these determinants and shows that the risk of mortality is higher than expected in groups of people in which different axes of inequality converge. The clearest example of this is the group of rural male migrants with more than 15 years in Spain and low annual income. This intersectional analysis reminds us of the importance of global perspectives to improve health equity problems.

## Additional material


Online Supplementary Document
