# Supplementary material for: Intersectional analysis of social determinants of health and their association with mortality in patients with multimorbidity
Source: J Glob Health. 2024 Oct 18;14:04229. doi: 10.7189/jogh.14.04229 (PMC11487491; doi:10.7189/jogh.14.04229)
Supplement: Online Supplementary Document [file jogh-14-04229-s001.pdf]

Table 1S: List of 129 chronic conditions analysed for the definition of multimorbidity based on the use of the Clinical Classifications Software (CCS) and the Chronic Condition Indicator Software.

CCS code	Condition
D1	Tuberculosis
D4	Mycoses
D5	HIV infection
D6	Hepatitis
D7	Viral infection
D8	Other infections; including parasitic
D48	Thyroid disorders
D51	Other endocrine disorders
D52	Nutritional deficiencies
D53	Disorders of lipid metabolism
D54	Gout and other crystal arthropathies
D56	Cystic fibrosis
D57	Immunity disorders
D59	Deficiency and other anemia
D61	Sickle cell anemia
D62	Coagulation and hemorrhagic disorders
D63	Diseases of white blood cells
D650	Adjustment disorders
D651	Anxiety disorders
D652	Attention-deficit, conduct, and disruptive behavior disorders
D653	Delirium, dementia, and amnestic and other cognitive disorders
D654	Developmental disorders
D655	Disorders usually diagnosed in infancy, childhood, or adolescence
D657	Depression and mood disorders
D658	Personality disorders
D659	Schizophrenia and other psychotic disorders
D660	Alcohol-related disorders

D661	Substance-related disorders
D670	Miscellaneous mental health disorders
D78	Other CNS infection and poliomyelitis
D79	Parkinson`s disease
D80	Multiple sclerosis
D81	Other hereditary and degenerative nervous system conditions
D82	Paralysis
D83	Epilepsy; convulsions
D84	Headache; including migraine
D86	Cataract
D87	Retinal detachments; defects; vascular occlusion; and retinopathy
D88	Glaucoma
D89	Blindness and vision defects
D90	Inflammation; infection of eye (except that caused by tuberculosis or sexually transmitted disease)
D91	Other eye disorders
D93	Conditions associated with dizziness or vertigo
D94	Other ear and sense organ disorders
D95	Other nervous system disorders
D96	Heart valve disorders
D97	Peri-; endo-; and myocarditis; cardiomyopathy (except that caused by tuberculosis or sexually transmitted disease)
D100	Acute myocardial infarction sequelae
D103	Pulmonary heart disease
D104	Other and ill-defined heart disease
D105	Conduction disorders
D106	Cardiac dysrhythmias
D107	Cardiac arrest and ventricular fibrillation
D108	Congestive heart failure; nonhypertensive
D109	Acute cerebrovascular disease sequelae
D111	Other and ill-defined cerebrovascular disease
D112	Transient cerebral ischemia
D113	Late effects of cerebrovascular disease
D114	Peripheral and visceral atherosclerosis

D115	Aortic; peripheral; and visceral artery aneurysms
D116	Aortic and peripheral arterial embolism or thrombosis
D117	Other circulatory disease
D121	Other diseases of veins and lymphatics
D127	Chronic obstructive pulmonary disease and bronchiectasis
D128	Asthma
D132	Lung disease due to external agents
D133	Other lower respiratory disease
D134	Other upper respiratory disease
D137	Diseases of mouth; excluding dental
D138	Esophageal disorders
D144	Regional enteritis and ulcerative colitis
D146	Diverticulosis and diverticulitis
D149	Biliary tract disease
D151	Other liver diseases
D152	Pancreatic disorders (not diabetes)
D155	Other gastrointestinal disorders
D156	Nephritis; nephrosis; renal sclerosis
D158	Chronic kidney disease
D159	Urinary tract infections
D162	Other diseases of bladder and urethra
D163	Genitourinary symptoms and ill-defined conditions
D164	Hyperplasia of prostate
D165	Inflammatory conditions of male genital organs
D166	Other male genital disorders
D167	Nonmalignant breast conditions
D169	Endometriosis
D170	Prolapse of female genital organs
D171	Menstrual disorders
D173	Menopausal disorders
D174	Female infertility
D175	Other female genital disorders

D183	Hypertension complicating pregnancy; childbirth and the puerperium
D197	Skin and subcutaneous tissue infections
D198	Other inflammatory condition of skin
D199	Chronic ulcer of skin
D200	Other skin disorders
D201	Infective arthritis and osteomyelitis (except that caused by tuberculosis or sexually transmitted disease)
D202	Rheumatoid arthritis and related disease
D203	Osteoarthritis
D204	Other non-traumatic joint disorders
D205	Spondylosis; intervertebral disc disorders; other back problems
D206	Osteoporosis
D208	Acquired foot deformities
D209	Other acquired deformities
D210	Systemic lupus erythematosus and connective tissue disorders
D211	Other connective tissue disease
D212	Other bone disease and musculoskeletal deformities
D213	Cardiac and circulatory congenital anomalies
D214	Digestive congenital anomalies
D215	Genitourinary congenital anomalies
D216	Nervous system congenital anomalies
D217	Other congenital anomalies
D225	Joint disorders and dislocations; trauma-related
D227	Spinal cord injury
D247	Lymphadenitis
D248	Gangrene
D252	Malaise and fatigue
D253	Allergic reactions
D259	Residual codes; unclassified
D300	Obesity
D301	Other microcrystalline arthritis
D302	Eating disorders
D303	Sexual disorders

D304	Sleeping disorders
D305	Somatization and hypochondria disorders
G2	Neoplasms
G3_23	Diabetes Mellitus
G7_1	Hypertension
G3_11	Other nutritional; endocrine; and metabolic disorders

Table 2S: Chronic diseases of patients with multimorbidity that died during the study period

MEN		WOMEN	
**Disease**	**N (%)**	**Disease**	**N (%)**
Hypertension	20,106 (64.4)	Hypertension	23,218 (72.7)
Disorders of lipid metabolism	12,828 (41.1)	Disorders of lipid metabolism	13,322 (41.7)
Diabetes Mellitus	9504 (30.4)	Genitourinary symptoms and ill-defined conditions	12,492 (39.1)
Other nutritional; endocrine; and metabolic disorders	7841 (25.1)	Osteoarthritis	11,375 (35.6)
Hyperplasia of prostate	7445 (23.8)	Depression and mood disorders	9161 (28.7)
Osteoarthritis	6809 (21.8)	Delirium, dementia, and amnestic and other cognitive disorders	8478 (26.6)
Cardiac dysrhythmias	6143 (19.7)	Diabetes Mellitus	8084 (25.3)
Genitourinary symptoms and ill-defined conditions	5986 (19.2)	Other nutritional; endocrine; and metabolic disorders	7959 (24.9)
Cataract	5825 (18.7)	Osteoporosis	7547 (23.6)
Chronic obstructive pulmonary disease and bronchiectasis	5316 (17.0)	Cataract	6268 (19.6)
Neoplasms	5183 (16.6)	Cardiac dysrhythmias	5717 (17.9)
Delirium, dementia, and amnestic and other cognitive disorders	5002 (16.0)	Thyroid disorders	5456 (17.1)
Coagulation and hemorrhagic disorders	4790 (15.3)	Anxiety disorders	5312 (16.6)
Depression and mood disorders	4472 (14.3)	Chronic kidney disease	4644 (14.6)
Chronic kidney disease	4209 (13.5)	Congestive heart failure; nonhypertensive	4569 (14.3)
Acute myocardial infarction sequelae	4194 (13.4)	Coagulation and hemorrhagic disorders	4248 (13.3)
Obesity	3629 (11.6)	Obesity	4224 (13.2)
Glaucoma	3552 (11.4)	Spondylosis; intervertebral disc disorders; other back problems	4150 (13.0)
Congestive heart failure; nonhypertensive	3547 (11.4)	Glaucoma	3994 (12.5)
Other ear and sense organ disorders	3462 (10.1)	Other ear and sense organ disorders	3571 (11.2)
Acute cerebrovascular disease sequelae	3126 (10.0)	Chronic ulcer of skin	3315 (10.2)

Spondylosis; intervertebral disc disorders; other back problems	3042 (9.75)	Neoplasms	3234 (10.1)
Gout and other crystal arthropathies	2923 (9.36)	Acute cerebrovascular disease sequelae	3033 (9.50)
Anxiety disorders	2888 (9.25)	Transient cerebral ischemia	2568 (8.05)
Transient cerebral ischemia	2134 (6.84)	Acute myocardial infarction sequelae	2463 (7.72)
Chronic ulcer of skin	2129 (6.82)	Asthma	2086 (6.54)
Thyroid disorders	2031 (6.51)	Blindness and vision defects	1873 (5.87)
Blindness and vision defects	1970 (6.31)	Chronic obstructive pulmonary disease and bronchiectasis	1628 (5.10)
Peripheral and visceral atherosclerosis	1598 (5.12)	Diverticulosis and diverticulitis	1390 (4.35)
Parkinson`s disease	1248 (4.00)	Retinal detachments; defects; vascular occlusion; and retinopathy	1268 (3.97)
